# Gamification as a Sustainable Source of Enjoyment During Balance and Gait Exercises

**DOI:** 10.3389/fpsyg.2019.00294

**Published:** 2019-03-01

**Authors:** Katinka van der Kooij, Rosanne van Dijsseldonk, Milou van Veen, Frans Steenbrink, Coen de Weerd, Krista E. Overvliet

**Affiliations:** ^1^Department of Human Movement Sciences, Vrije Universiteit Amsterdam, Amsterdam, Netherlands; ^2^Department of Research, Sint Maartenskliniek, Nijmegen, Netherlands; ^3^Motekforce Link, Amsterdam, Netherlands; ^4^Department of Biological Psychology and Neuropsychology, University of Hamburg, Hamburg, Germany; ^5^Department of Experimental Psychology, Utrecht University, Utrecht, Netherlands

**Keywords:** motivation, pleasure, reward, psychomotor performance, video games, postural balance, gait, exercise

## Abstract

We may be motivated to engage in a certain motor activity because it is instrumental to obtaining reward (e.g., money) or because we enjoy the activity, making it intrinsically rewarding. Enjoyment is related to intrinsic motivation which is considered to be a durable form of motivation. Therefore, many rehabilitation programs aim to increase task enjoyment by adding game elements (“gamification”). Here we ask how the influence of game elements on motivation develops over time and additionally explore whether enjoyment influences motor performance. We describe two different studies that varied game elements in different exercises. Experiment 1 compared the durability of enjoyment for a gamified and a conventional balance exercise in elderly. Experiment 2 addressed the question whether adding game elements to a gait adaptability exercise enhances the durability of enjoyment and additionally tested whether the game elements influenced movement vigor and accuracy (motor performance). The results show that the game elements enhanced enjoyment. Enjoyment faded over time, but this decrease tended to be less pronounced in gamified exercises. There was no evidence that the game elements affected movement vigor or accuracy.

## Introduction

Fred and Frank climb the Mount Everest. Fred is motivated for the hike because he will receive sponsor money when he reaches the top. Frank is motivated because he enjoys the activity of hiking. In other words: Fred is motivated by an *extrinsic* reward that is separable from the activity of hiking whereas Frank is motivated by an *intrinsic* reward that is inherent to the activity of hiking (Deci and Cascio, 1972). Task enjoyment is associated with intrinsic motivation (Ryan and Deci, 2017) which is considered a durable form of motivation that doesn’t decrease much over time (Vansteenkiste et al., 2006). Consequently, there is a lot of enthusiasm about enhancing enjoyment in the context of rehabilitation. Moreover, enjoyment is an intrinsic form of reward and research in the field of motor learning has shown that rewards can enhance motor performance (Cheng et al., 2013; Reppert et al., 2015; Shadmehr et al., 2016; Summerside et al., 2018). Would Frank’s enjoyment not only be durable but also influence how he performs the hike? In this paper, we investigate whether enjoyment of a rehabilitation exercise can be enhanced in a durable manner by adding game elements to the exercise, a process called “gamification” (Deterding et al., 2011) also known as “serious game” design or “persuasive game” design (Visch et al., 2013).

One reason why gamification has the power to enhance enjoyment is that it can the fulfill the basic psychological needs of autonomy, competence and relatedness described by Self Determination Theory (Ryan and Deci, 2000). Game worlds can foster Autonomy by setting aside “real-world” limitations. Moreover, digital games can offer a greater level of variety and player options and choice (Ryan and Deci, 2017). Offering free choice enhances experienced autonomy (Wulf et al., 2014). Similarly, the need for Self-Competence can be fulfilled by balancing of game difficulty and player skill (“flow”) and by providing direct feedback on performance (Ryan et al., 2006; Przybylski et al., 2010). Gamification is thus suitable to enhance enjoyment. But before using it to increase the amount of exercise, it is important to understand the temporal development of the enjoyment.

The enjoyment may be durable because the psychological needs do not become satiated like physiological needs (Deci and Ryan, 2000). However, the extent to which an activity fulfills psychological needs may change over time. As a child, Frank may have felt competent walking to the park but as he has aged this activity probably no longer fulfilled his need for self-competence. Moreover, novelty has been proposed as an important cause of intrinsic motivation (White, 1959). If novelty is important, enjoyment may fade while novelty wears off. Indeed, the motivating influence of gamification has been found to decrease over time. In one study, school children’s intrinsic motivation for performing video game exercises (exergames) during physical education faded over time (Sun, 2013). In another study use of a set of exergames decreased dramatically over time (Simons et al., 2015). What remains unknown is whether adding game elements, enhances the durability of enjoyment.

A second issue we raise here is whether Frank’s enjoyment would influence how he performs the hike. There are two ways in which enjoyment may influence motor performance: by influencing movement vigor and by influencing movement accuracy. For extrinsic rewards such as money or food it is well established that they affect movement vigor (Shadmehr et al., 2016). Humans (Cheng et al., 2013; Reppert et al., 2015) make faster eye movements toward more rewarding stimuli. Similarly, reaching movements are faster toward more preferred candy (Sackaloo et al., 2015). In addition, one study showed that financial reward enhanced the accuracy of pointing movements under perturbed feedback (Gajda et al., 2016). One study even showed that reward can break the speed-accuracy trade-off (Fitts, 1954) making movements faster *and* more precise (Manohar et al., 2015). For intrinsic rewards such as those associated with enjoyment, their effect on motor performance is less clear. A few studies showed that positive feedback enhanced movement accuracy (Jourden et al., 1991; Bandura, 1997; Lewthwaite and Wulf, 2010). For instance, repeatedly telling participants that they performed above average - reduced sway in a balance task (Lewthwaite and Wulf, 2010). Also, framing a task as one that could be learnt instead of framing it as one that measures a basic ability improved performance (Jourden et al., 1991) and time-on-target in a pursuit rotor task (Bandura, 1997). Other studies report that performance was not enhanced by visual and audio design (Lohse et al., 2015), free choice of ball color (Wulf et al., 2014), or positive feedback resulting from loose success criteria (Ong and Hodges, 2017).

In the current paper, we describe two experiments that address the question whether game elements enhance the level and durability of enjoyment. Experiment 1 compared enjoyment between a commercially available gamified balance exercise a and conventional balance exercise. Experiment 2 compared enjoyment between a commercially available gait adaptability exercise and the same exercise with a number of game elements removed. In addition, Experiment 2 assessed the influence of game elements on movement vigor and accuracy. The experiments were not originally designed as parts of a single study. Because they do tell an overall story, they are presented together in this paper.

## Experiment 1: Gamified Versus Conventional Balance Exercise: Intrinsic Motivation

Experiment 1 addressed the question whether gamification enhances the level and durability of enjoyment of balance exercise in elderly and tested three hypotheses. First, we hypothesized that the game elements would enhance enjoyment. Enjoyment probably decreases over time as motivation for exergames decreases over time (Sun, 2013; Simons et al., 2015). We therefore hypothesized that enjoyment decreases over time. As game elements induce enjoyment by fulfilling psychological needs that do not satiate (Ryan et al., 2006), we critically predict that game elements enhance the durability of enjoyment.

### Methods

#### Design

We used a between-subjects design in which we, in a pseudo-random order assigned half of the participants to the game group and the other half to the control group.

#### Participants

As the investigated balance training was designed for elderly users, participants (*N* = 28) were healthy community dwelling elderly (19 women and 9 men, age: 75.2 ± 6.6 years). Two participants dropped out during the study and were not included in the final analysis. Eventually, 12 participants took part in the game group and 12 participants took part in the control group. Participants were recruited from fitness classes offered for elderly and via the personal network of the experimenter. Inclusion criteria were: age 65 years or older, the ability to walk safely without assistance and/or assistive devices. Exclusion criteria were: neurological disorder, cardiopulmonary disorder and impaired vision after correction.

We used a single-blind parallel trial design in which participants from both groups were told that they would participate in a study comparing the efficacy of two types of balance training. Participants were recruited at fitness centers offering fitness classes for elderly. Participants who were recruited from the same fitness facility were assigned to the same experimental condition to keep participants blind to the experimental conditions. Duo’s were allocated to one of the two experimental conditions in a random order with an allocation ratio of 1:1. Participants were tested at four locations in Netherlands: a fitness center in Utrecht, a community center in Utrecht and a community center in Graft and a second fitness center in Utrecht. Participants were tested at the location that was closest to where they were recruited.

The study was carried out in accordance with the recommendations of the Declaration of Helsinki and the ethical committee of the Faculty of Behavioural and Human Movement Sciences at the Vrije Universiteit Amsterdam. The protocol was approved by the ethical committee of the Faculty of Behavioural and Human Movement Sciences at the Vrije Universiteit Amsterdam. All subjects gave written informed consent in accordance with the Declaration of Helsinki.

#### Materials Experiment 1

Prior to starting the experimental test sessions participants were informed that they would participate in a study on balance training with the following text:

“Balance is essential to many daily activities. Balance declines with age and it can therefore be important to engage in balance training. The purpose of this study is to compare the effectivity of two different balance trainings.”

**FIGURE 1:**
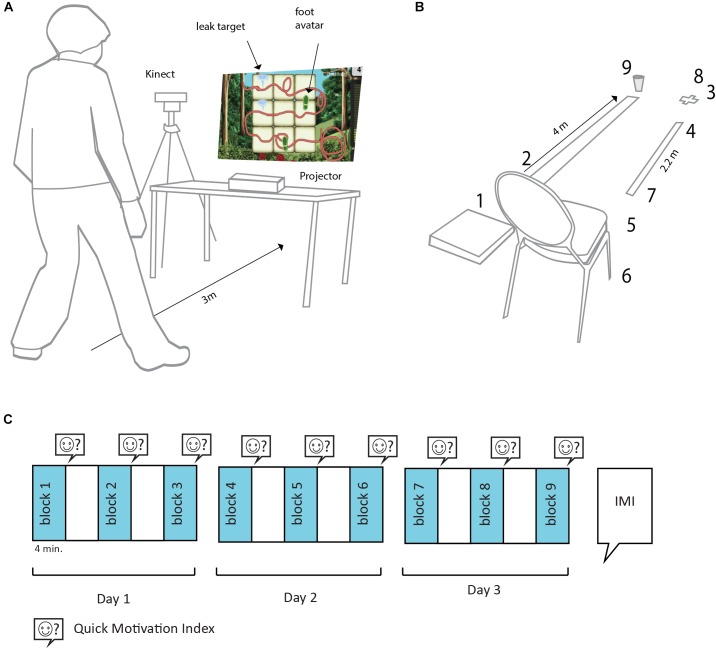
Methods Experiment 1. **(A)** Gamified balance exercise with the “garden hose” game. **(B)** Conventional balance course: (1) Stepping on a foam cushion. (2) Walking along a narrow path. (3) Turning around full cycle. (4) Walking backward. (5) Standing up from a chair with arms crossed over the chest. (6) Single leg stance. (7) Tandem walking. (8) Single-leg swing. (9) Lifting a glass of water, walking while holding it, and placing it back on the ground. **(C)** Sequence of tasks and assessments.

The game group performed the “Garden hose game” which is a balance exercise designed by SilverFit BV that challenged the participant to maintain balance while placing the feet in varying positions. The game employed a screen and projector for visual display and a Kinect v2 for motion registration such that direct feedback on the participant’s movements could be provided (Figure 1A). The screen displayed a garden hose in which two simultaneous target leaks could appear, together with a representation of the participant’s feet. The task of the participant was to seal both leaks by placing a foot on each of them. When a leak had been sealed, the participant scored points, and two new leaks appeared. Game elements involved direct feedback on the stepping movements, scores for successful performance, audio and graphic design and a game narrative of sealing leaks in a garden hose. The exercise ended after 4 min.

The control group performed a conventional balance course described in the literature as suitable for the target group (Pluchino et al., 2012). The balance course offered the following tasks in a fixed order (Figure 1B): (1) stepping on a compliant surface (the Sportbay ^®^Balance Pad). (2) Walking over a narrow path indicated on the floor. (3) Turning around full cycle. (4) Backward walking. (5) Standing up from a chair with arms crossed over the chest. (6) Single leg stance. (7) Tandem walking. (8) Single leg swing. (9) Lifting a glass of water from the floor, walking while holding it, and placing it back on the ground. The balance course ended after 4 min. If participants completed the course within 4 min a second round started.

Enjoyment was assessed using the Interest/Enjoyment scale of the Intrinsic Motivation Inventory (IMI, Ryan, 1982) and using a “Quick Motivation Index” (QMI) developed for the experiment. The English version of the IMI has adequate internal-consistency (α = 0.85) (McAuley et al., 1989). We translated this version to Dutch to make it suitable for the elderly population. On the pen and paper questionnaire, participants indicated their agreement with several statements on a 7-point Likert scale. The QMI consisted two-items ratings in which participants were asked to respond vocally to the following two questions:

(1) On a scale of one to ten how much do you enjoy the task until now?
(2) On a scale of one to ten how motivated are you to continue?

#### Procedure Experiment 1

Participants were informed that balance exercises were repeated on three different days with a minimum interval of 1 week (Figure 1C) and that each session would take about 24 min, consisting of three 4-min exercise blocks interleaved by 4 min rest. Experimental sessions were performed in groups of two participants from the same experimental condition (game or control). The members of the duo alternately exercised and rested. Following each block participants responded to the two-item motivation ratings. At the end of the final session, participants completed the IMI.

#### Data Analysis Experiment 1

To assess the agreement between the QMI and the IMI as an indicator of the level of enjoyment, a Spearman rank-order correlation was calculated between the IMI Interest/Enjoyment subscale and QMI ratings. To further assess test-retest reliability of the QMI we calculated the intra-class correlation between the three test days for each block. Data analyses were aimed at testing two hypotheses, concerning the influence of gamification on enjoyment and concerning the temporal development of enjoyment.

(1) To test the hypothesis that a gamified exercise is more enjoyable than a conventional exercise, the QMI scores averaged over blocks were compared between the game and control group using a Mann-Whitney *U*-test.
(2) To test the hypothesis that enjoyment decreases over time we performed a Friedman rank order test on the QMI in the different blocks.
(3) To test the hypothesis that enjoyment was more durable in the game group than in the control group, we assessed whether the change in QMI from block 1 to 9 (ΔQMI = QMI_block__9_ – QMI_block__1_) was less negative for the game group compared to the control group using a one-sided Mann-Whitney *U*-test.

### Results Experiment 1

**FIGURE 2:**
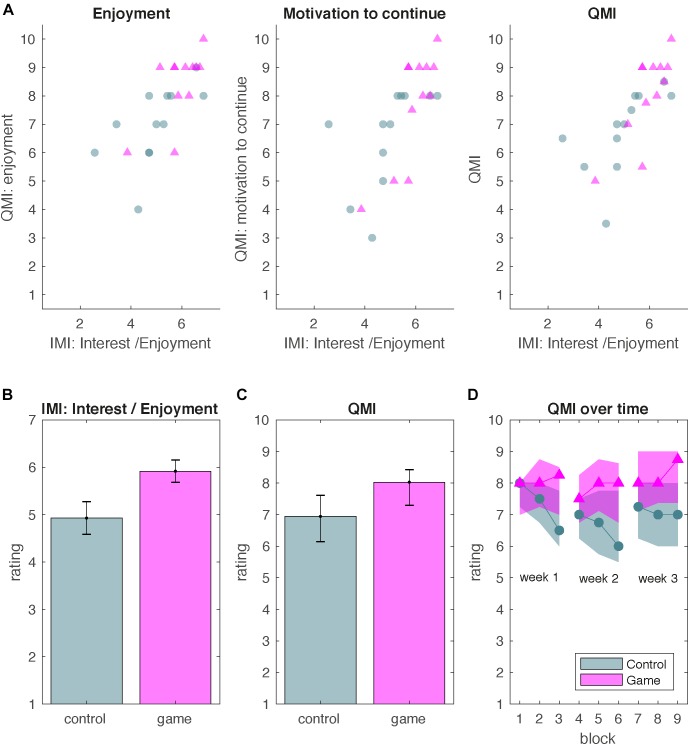
Results Experiment 1. Intrinsic motivation for balance training. Green circles represent the control group and pink triangles represent the game group. **(A)** IMI Interest/Enjoyment subscale rating as a predictor of responses on the enjoyment rating, motivation-to-continue rating and Quick Motivation Index (QMI). **(B)** Mean IMI scores for the game and control group. Error bars represent standard errors of the mean. **(C)** Median QMI scores for the game and control group. Error bars represent the interquartile range. **(D)** Median QMI as a function of block for the game and control group. Shaded areas represent the interquartile range.

The Interest/Enjoyment subscale was positively correlated with the rating of enjoyment (*r* = 0.75, *p* < 0.001), rating of motivation to continue (*r* = 0.77, *p* < 0.001) and QMI (*r* = 0.81, *p* < 0.001; Figure 2A). Moreover, a good reliability was found between the QMI measurements on different days. The average measure ICC was 0.83 with a 95% confidence interval from 0.66 to 0.92 for block 1, 0.84 with a 95% confidence interval from 0.69 to 0.93 for block 2 and 0.92 with a 95% confidence interval from 0.84 to 0.96 for block 3.

The game group scored higher on the IMI than the control group [*t*(22) = -2.37, *p* = 0.03; Figure 2B]. The total QMI score over all blocks did not differ significantly between the game and control group (*U* = 40.5, *z* = 1.82, *p* = 0.068).

The durability of enjoyment was higher for the game group than for the control group (*U* = 38, *z* = -1.99, *p* = 0.046; Figure 2C). To analyze whether motivation changed in both groups, we performed two additional Friedman tests on the QMI in the control and game group (*χ*^2^ = 29.04, *p* < 0.001). In the control group, the QMI decreased over repeated blocks by 13% (*χ*^2^ = 25.88, *p* < 0.001). In the game group, in contrast, the QMI *increased* by 6% over repeated blocks (*χ*^2^ = 22.85, *p* < 0.001).

*Post hoc* power analyses showed that statistical power for the Mann-Whitney *U*-tests was low (0.64 for detecting a large effect size).

Thus, Experiment 1 showed that the QMI correlates with the Interest/Enjoyment subscale of the IMI. Most importantly, Experiment 1 showed that gamification affects the durability of enjoyment. In the control group, enjoyment decreased over time whereas in the game group enjoyment increased.

## Experiment 2: Added Game Elements in Gait Adaptability Exercise: Intrinsic Motivation and Performance

Experiment 2 addressed the question whether adding game elements to a gait adaptability exercise enhances the level and durability of exercise enjoyment. We hypothesized that game elements render a task more enjoyable and that enjoyment decreases over time. Because game elements create enjoyment by tapping into psychological needs that do not satiate (Ryan et al., 2006), we tested the hypothesis that game elements enhance the durability of enjoyment. In addition, based on the finding that rewards enhance movement vigor and perhaps also movement accuracy (Cheng et al., 2013; Reppert et al., 2015; Sackaloo et al., 2015; Shadmehr et al., 2016; Summerside et al., 2018) we explored whether the game elements enhanced movement vigor and accuracy.

### Methods Experiment 2

#### Design

We used a parallel, single-blind trial in which, in a random order, half of participants was assigned to the game group and the other half to the control group. Participants were blinded to the experimental hypothesis by informing them that they would participate in a study investigating the effect of virtual feedback on gait rehabilitation.

#### Participants

We recruited forty-two healthy adults, mainly students Human Movement Sciences at the Vrije Universiteit (23 females and 19 males, age: 24.5 ± 8.37 years). Exclusion criteria were people who had experienced injuries that could affect their walking stability, who could not walk continuously for 30 min, and who had received treadmill-walking therapy in the past or were familiar with Motekforce Link’s gait adaptability game (Microbes). This study was carried out in accordance with the recommendations of the Declaration of Helsinki and the ethical committee of the Faculty of Behavioural and Human Movement Sciences at the Vrije Universiteit Amsterdam. The protocol was approved by the ethical committee of the Faculty of Behavioural and Human Movement Sciences at the Vrije Universiteit Amsterdam. All subjects gave written informed consent in accordance with the Declaration of Helsinki. Participants were tested at the Motekforce Link office in Amsterdam.

#### Materials

**FIGURE 3:**
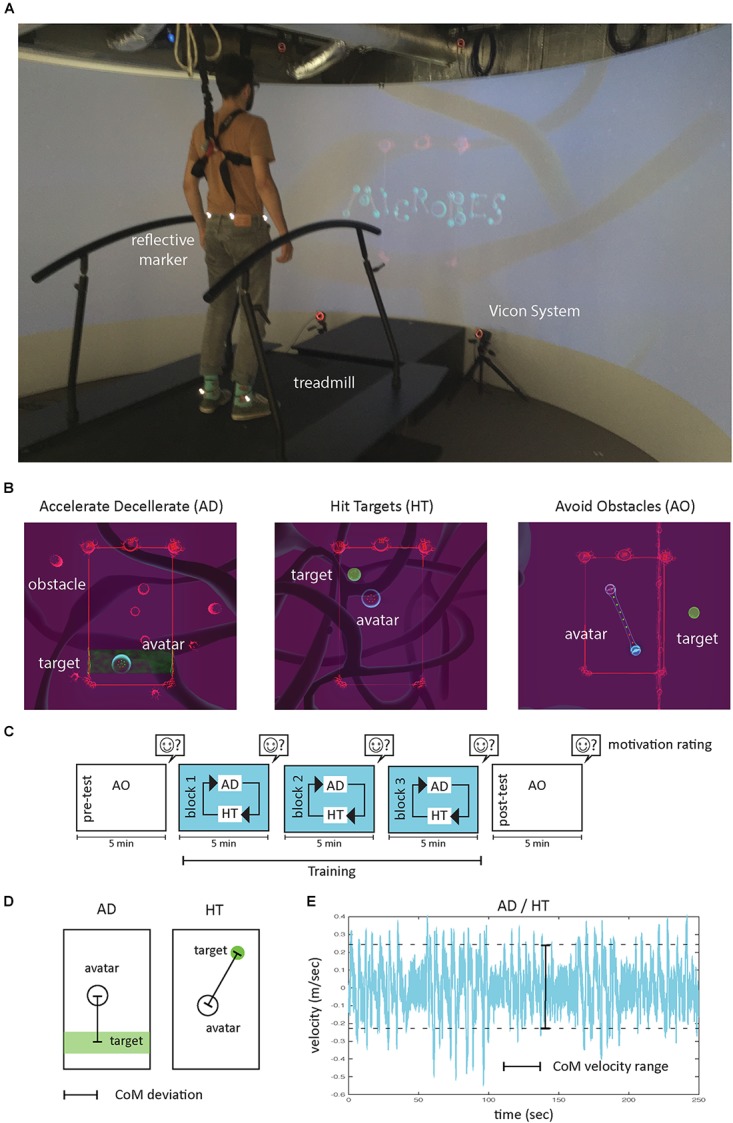
Methods Experiment 2. **(A)** Gait Real-time Interactive Lab (GRAIL) system with treadmill, screen and Vicon system. **(B)** Microbes mini games with avatar, target and obstacles. **(C)** Sequence of tasks and assessments. **(D)** Movement accuracy was measured by the center of mass (CoM) deviation: the distance between the CoM avatar and target. **(E)** Movement vigor was measured from the 95% velocity range of the CoM avatar during a block.

The gait adaptability exercise was developed by Motekforce Link for the Gait Real-time Analysis Interactive Lab (GRAIL; Figure 3A). In the exercise participants walked on a treadmill while a virtual environment projected in front of them challenged them to catch virtual targets and avoid virtual obstacles that moved into the playing field from a random location outside the field. The targets could be caught and avoided by controlling the position of an avatar with the participant’s center of mass (CoM) as tracked by the GRAIL system (Motekforce Link; for a video impression see^1^). The Microbes game is rich in game elements, taking advantage of the virtual environment to immerse the participant in a visually engaging fantasy world, balancing challenges to player skill, providing direct feedback using both audio and graphic effects and providing game scores. To ensure that the game challenged the healthy participants, we set the treadmill speed 10% above the preferred walking speed (Hak et al., 2012). The Microbes game consists of several minigames of which we selected three mini games (Figure 3B). The “Avoid Obstacles” (AO) mini game (Figure 3B; sec 4.20 in the video impression) was selected for a motivation pre- and post-test. The ”Acceleration/Deceleration” (AD) mini game (sec 2.50 in the video impression), and Hit targets (HT) mini game (sec 1 in the video impression) were selected for the main experiment. For both the AD and HT mini game a control game was created from which all game elements that were expected to affect motivation but who had no direct influence on motor performance were removed. The following game elements were removed: visual display of scores, audio design, graphic design, visual display of the remaining time, number of caught targets, and the game narrative of an evolving Microbe.

Enjoyment was measured using the QMI that we also used in Experiment 1. The only difference being that we used a 7-point scale instead of a 10-point scale. Experiment 1 used a wider scale because this experiment was performed after Experiment 2 and aimed to achieve a higher QMI precision.

#### Procedure

The procedure is schematically depicted in Figure 3C. First, the participant’s preferred walking speed was determined. For each participant the maximal comfortable treadmill walking speed (MCTWS) was determined based on a 1-up, 1-down staircase procedure with a step-size of 0.1 m per second described in detail by Dal et al. (2010). Next, participants were informed that they would perform a walking task that would last in total about 30 min. They were also reminded that during the task they should remember that green objects (the targets) were good whereas red objects (the obstacles) were evil. No other instructions were provided. After that they performed the 5-min AO minigame pre-test. When the pre-test was finished, we explained the game narrative of an evolving Microbe to the participants in the game group: “You will be a microbe in a world of other bacteria and organisms. You live in a fictional world in which you need to collect life essence. Each time you have collected a life essence you are closer to accomplishing your ultimate goal: developing and reaching the next level!”

After that, participants performed three 5-min blocks in which the AD and HT mini game alternated every time seven targets were caught within a game. After each block, the treadmill was stopped and the QMI was administered. After three blocks, another “AO” mini game was performed as a motivational post-test.

#### Data Collection and Analysis

Kinematic data on movement vigor and accuracy were based on the 2D position of the CoM avatar that the participant controlled with his or her center of mass. To this end, the CoM was tracked with the Vicon (Oxford, United Kingdom) motion capture system embedded in the GRAIL system and the x, y position on the treadmill was calculated by the direct average of four reflective markers placed on the pelvis. Kinematic data were filtered offline in MATLAB 2017b using a two-way second-order Butterworth filter with a cut-off frequency of 2 Hz and were also analyzed using MATLAB.

*Movement vigor* was analyzed by the CoM avatar’s *velocity range* (*Vrange*, Figure 3E), which was the 95% range of the velocity of the CoM avatar within a block. This value reflects the range in which participants accelerated or decelerated in order to catch the targets and avoid the obstacles while the treadmill imposed a velocity that was constant.

*Movement accuracy* was measured from the *CoM deviation* (Figure 3D), which was the mean distance between the position of the CoM avatar and target position within a block. In the AD mini game, movement accuracy was calculated as the absolute one-dimensional distance between the y position of the CoM avatar and the middle of the target area. In the HT minigame, the movement accuracy was the absolute two-dimensional distance (x, y position) between the CoM avatar and the center of the target circle.

Statistical tests were performed with SPSS version 24 and focused on 4 hypotheses testing the influence of game elements on motivation and testing the influence of motivation on movement vigor and movement accuracy:

Motivation

(1) To test the hypothesis that adding game elements increases task enjoyment, the overall QMI score was compared between the game and control group using a Mann-Whitney *U*-test.
(2) To test the hypothesis that adding game elements enhances the durability of enjoyment we tested whether the change in QMI between block 1 and 3 (ΔQMI) was more negative for the control group compared to the game group using a one-sided Mann-Whitney *U*-test.

Movement vigor

(3) To test whether game elements influenced the movement vigor, we entered the CoM velocity range in the three blocks in a mixed ANOVA with group as a between-participants factor and block as a repeated factor.

Movement accuracy

(4) To test whether game elements influenced movement accuracy we entered the CoM deviation data in the three blocks in a mixed ANOVA with group as a between participants factor and block as a repeated factor.

### Results Experiment 2

#### Enjoyment

At pre-test, the QMI did not differ between groups, *U* = 373, *z* = -1.58, *p* = 0.11. Neither did the QMI differ between groups at post-test, *U* = 391, *z* = -1.06, *p* = 0.29. This indicates that differences between groups were caused by the difference in game elements during the three experimental blocks.

**FIGURE 4:**
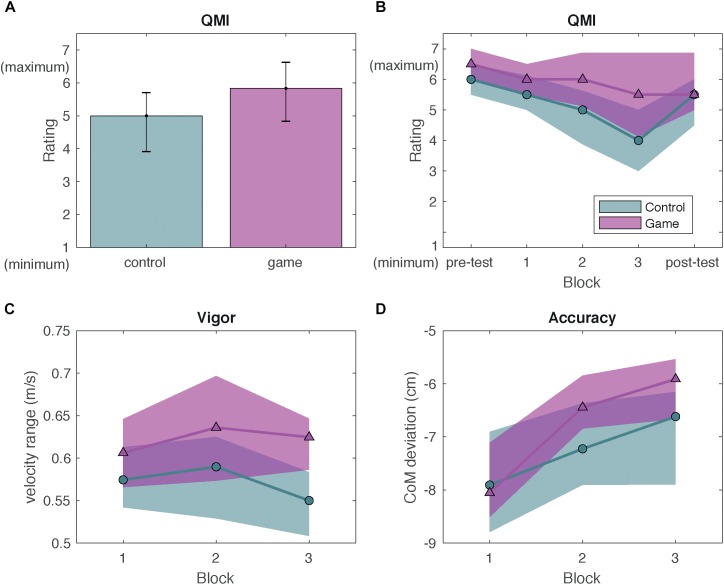
Results Experiment 2. Green circles represent data from the control group and pink triangles represent data from the game group. Shaded areas indicate the interquartile range. **(A)** Median QMI for the game and control group. Error bars represent the interquartile range. **(B)** Median QMI with interquartile range as a function of block. **(C)** Median movement vigor with interquartile range as a function of block. **(D)** Median movement accuracy with interquartile range as a function of block.

The Mann-Whitney *U*-test comparing the QMI averaged over the three experimental blocks between the game and control group showed that enjoyment was higher in the game group (Median = 6) compared to the control group (Median = 5), *U* = 104, *z* = -2.59, *p* = 0.01 (Figure 4A). The Friedman test comparing the QMI in block 1, 2, and 3 showed that the QMI decreased over blocks by 18%, *χ*^2^ = 38.26, *p* < 0.001. However, the decrease in enjoyment (ΔQMI) did not significantly differ between the game and control group, *U* = 138.5, *z* = -1.67, *p* = 0.095. Figure 4B does show a pattern consistent with Experiment 1: the decrease was less pronounced in the game group. *Post hoc* power analyses showed that statistical power for the Mann-Whitney *U*-tests was adequate only to detect a large effect size (power was 0.8 for detecting a large effect size).

#### Movement Vigor

The mixed ANOVA on the CoM velocity range in the game and control group (Figure 4C) showed no main effect of group, *F*(1,37) = 0.48, *p* = 0.49, no interaction of group and block, *F*(2,74) = 1.16, *p* = 0.32 and no main effect of block, *F*(2,74) = 1.99, *p* = 0.14. Thus, movement vigor did not differ between the game and control group.

#### Movement Accuracy

The mixed ANOVA on the CoM distance (Figure 4D) as a between-participants factor and with block as a repeated factor showed a main effect of block, *F*(1.34,49.57) = 15.46, *p* < 0.001, no main effect of group *F*(1,37) = 2.32, *p* = 0.14 and no interaction of group and block *F*(1.34,49.57) = 0.21, *p* = 0.72. Thus, there was no evidence that movement accuracy was higher in the game group.

Thus, Experiment 2 showed that gamification enhanced enjoyment. Although the data on the durability of enjoyment showed a pattern similar to Experiment 1, there was no significant effect of gamification on the durability of enjoyment. Game elements did not influence movement vigor or accuracy.

## General Discussion

This study investigated how the enjoyable effect of game elements on movement exercises develops over time. Are game elements an inexhaustible source of intrinsic reward or does enjoyment fade over time? Additionally, we also explored whether game elements enhance movement vigor and accuracy, as has been found for extrinsic reward (Haggard et al., 2000; Takikawa et al., 2002; Opris et al., 2011; Cheng et al., 2013; Manohar et al., 2015; Reppert et al., 2015; Sackaloo et al., 2015; Gajda et al., 2016). Overall, we found that the game elements increased enjoyment. Enjoyment, as measured with the QMI, decreased while participants repeatedly performed the same exercise (Experiment 1 and 2). However, in Experiment 1, enjoyment *increased* for participants who performed the gamified exercise. In Experiment 2 the decrease in motivation appeared to be attenuated for the game group, but there was no significant difference between the game and control group. There was no evidence that the game elements enhanced movement vigor or accuracy. In sum, our main finding is that gamification enhances the level and probably the durability of enjoyment.

### Limitations

Before discussing the results any further, we need to emphasize two important limitations of the studies reported in this paper. First, we used a non-validated scale (QMI) that we developed for the experiments to measure the temporal decay of enjoyment. In addition, Experiment 1 used a non-validated Dutch Translation of the IMI. Therefore it remains to be established how the QMI relates to the construct of intrinsic motivation. A promising finding was that correlations between the QMI and the translated Interest/Enjoyment subscale of the IMI were adequate as were test-retest correlations for the QMI. Second, no *a priori* power analysis was performed, and sample sizes were only adequate to detect large effect sizes. For the influence of gamification on the level of enjoyment, the data were consistent across experiments, which adds evidence to the conclusion that the gamification increased the level and probably also the durability of intrinsic motivation. However, the finding that there was no relation between enjoyment and motor performance is difficult to interpret. Thus, the results should be read as preliminary evidence that can be used to design future studies.

### The Influence of Game Elements on Enjoyment

Our study confirms the idea that gamification can increase enjoyment of a task (for reviews see Connolly et al., 2012; Hamari et al., 2014). Experiment 1 showed that enjoyment of a balance exercise was higher for participants who performed a gamified exercise that involved sealing leaks with one’s footsteps than for participants who performed a conventional balance course consisting of a set of nine different tasks (see methods Experiment 1). As the movement tasks differed between the gamified and conventional training, the difference in enjoyment may have been related to the task rather than to the game elements in the gamified exercise (scores, aesthetic design, direct feedback, narrative). Experiment 2 tested whether the removal of game elements from a gamified gait adaptability exercise reduced enjoyment of this exercise. We found that the participants who performed the exercise without additional game elements (scores, aesthetic design, narrative) indeed experienced the exercise as less enjoyable.

Enjoyment can be considered an intrinsic form of reward that evokes motivation. Consistent with this idea, we measured enjoyment by asking both about enjoyment directly and by asking about motivation to continue and found that responses on the two items were highly correlated (Figure 2A). This suggests that when motivation needs to be enhanced, creating intrinsically rewarding games provides an alternative to using extrinsic rewards and punishments such as financial incentives.

A potential benefit of using gamification to enhance motivation is that it might stimulate an intrinsic type of motivation in which the motivation is perceived as internally regulated. The benefit of evoking intrinsic motivation is that intrinsic motivation has been associated with greater persistence at a task than extrinsic motivation in which the motivation is perceived as externally regulated (for a review see Deci et al., 1999). Moreover, intrinsic motivation has been proposed to result in higher quality performance than extrinsic motivation (Wulf and Lewthwaite, 2016; Ryan and Deci, 2017). Whereas intrinsic rewards foster intrinsic motivation, extrinsic rewards tend to reduce the intrinsic type of motivation (Deci et al., 1999; Ryan and Deci, 2017) although there has been some controversy on this subject (Cameron and Pierce, 1994). Thus, gamification may benefit the durability of motivation by increasing intrinsic motivation. Off course, gamification would only result in durable motivation if the enjoyment of the gamification is durable. In the next paragraphs we consider the temporal decay of enjoyment and whether there was a relation with motor performance.

### The Durability of Intrinsic Motivation

Using a novel QMI, we showed the expected decrease in enjoyment over time. Interestingly, for the gamified exercises enjoyment increased over time (Experiment 1) or the decrease tended to be attenuated (Experiment 2). This is a clear indication that game elements enhance the durability of enjoyment, as opposed to studies that show that motivation for exergames decreases over time (Sun, 2013; Simons et al., 2015). Game elements may enhance the durability of enjoyment because the enjoyment results from the fulfillment of needs that do not satiate (Ryan et al., 2006). In contrast to conventional exercise, game elements can create myriad opportunities for fostering these needs, for instance providing positive feedback to enhance competence and offering choice to foster autonomy. The attenuation of the decrease was statistically significant following the nine 5-min blocks of Experiment 1 but not following the three 5-min blocks of Experiment 2. Reported effects of rewards on motivation have been small (Rummel, 1988). It is thus well-possible that the absence of a statistically significant effect in Experiment 2 was due to a lack of statistical power. The longest timescale on which we assessed intrinsic motivation was 3 weeks. This is a much shorter timescale than the timescale on which intrinsic motivation is needed in for instance a rehabilitation or sports setting. How the influence of game elements on intrinsic motivation develops over longer timescales is an important question for future research.

### Intrinsic Motivation and Movement Vigor and Accuracy

Although we observed that game elements influenced the level of enjoyment, creating intrinsic reward, we did not find that they enhanced movement vigor or accuracy as has been shown for extrinsic reward (Haggard et al., 2000; Takikawa et al., 2002; Opris et al., 2011; Cheng et al., 2013; Manohar et al., 2015; Reppert et al., 2015; Sackaloo et al., 2015; Gajda et al., 2016). It is possible that we did not observe an effect of the gamification on movement vigor or accuracy because our study lacked the statistical power to detect such an effect. Effects of rewards on motor performance are often small, especially for intrinsic rewards such as scored points (Summerside et al., 2018) or faces (Xu-Wilson et al., 2009) and influences on learning are highly variable (Galea et al., 2015; van der Kooij and Overvliet, 2016; van der Kooij et al., 2018). It is to be expected that effects of enjoyment are similarly small. Moreover, measuring enjoyment with self-reports may lack sensitivity. Besides by using a larger sample size, future studies on the influence of enjoyment on motor performance could improve statistical power by increasing measurement sensitivity and by capitalizing on the passage of time as a factor that increases between-group differences in enjoyment.

### Suggestions for Future Research

To gain a better understanding of how and when gamification benefits real-world behavior, time sensitive measurements of outcome variables such as motivation need to be developed (van der Kooij et al., 2015). Using the novel QMI our study suggests that a benefit of gamification is that it enhances the durability of enjoyment and thereby intrinsic motivation. To our knowledge, no validated and suitable tool was available to assess enjoyment repeatedly without interfering with the gamified exercise. Although the results look promising, a number of methodological improvements may be made in the QMI. First, sensitivity and options for statistical analyses could be improved by collecting continuous rather than ordinal data. For instance by using a magnitude estimation protocol (Stevens, 1975). Second, we used verbal reports to avoid interference with the movement tasks. Validity of the QMI may be improved by using anonymous ratings that are incorporated in the game software instead of oral responses which may be biased by socially desirable answers. An interesting question for future research is whether including both a question on enjoyment and a question on motivation to continue provides an assessment of enjoyment-based intrinsic motivation or whether these two questions address different aspects of motivation.

Another interesting topic for future research is whether influences of gamification on enjoyment and motor performance differ between patients and healthy participants, such as tested in the current study. For instance, influences of gamification on enjoyment may be different because healthy participants and patients are motivated by different types of rewards. The healthy participants participated voluntarily. They may have intended to help the experimenter, contribute to science or may have been curious. Consistent with the relatively high baseline level of enjoyment for both the game and control group (Figure 2D, 4B), the healthy participants may have been largely motivated by rewards that are intrinsic to the activity of participating although participants in Experiment 2 may also have participated for the financial compensation. Patients, in contrast, are generally required to perform their rehabilitation exercises and may have be largely motivated by reward extrinsic to the exercise, for instance faster recovery. Influences of gamification on enjoyment may be larger for a group with a lower baseline enjoyment. Gamification may also affect motor performance differently for patients and healthy participants. Baseline levels of motor performance will be lower, perhaps allowing for a greater influence of gamification on motor performance (Deci et al., 1999; Eisenberger et al., 1999; Summerside et al., 2018).

## Conclusion

Adding game elements to a task enhances the level of enjoyment and causes the enjoyment to fade slower over time than it would otherwise. There was no evidence that enjoyment led to better motor performance. An interesting avenue for future research would be to develop paradigms that use the influence of time to study motor performance under varying levels of enjoyment. This way we can develop a deeper insight into whether Frank’s enjoyment would affect how he performs the hike.

## Author Contributions

KvdK, RvD, MvV, CdW, KO, and FS wrote the manuscript. KvdK, RvD, and MvV analyzed the data and collected the data. KvdK designed the Experiment 1. CdW, RvD, MvV, KO, and KK designed the Experiment 2.

## Conflict of Interest Statement

CdW and FS are employed by Motekforce Link, the company that developed the gamified gait adaptability exercise that we used in Experiment 2. The remaining authors declare that the research was conducted in the absence of any commercial or financial relationships that could be construed as a potential conflict of interest.

## Footnotes

1 https://www.youtube.com/watch?v=bgV2-Tb6OJQ
